# Gene expression responses of CF airway epithelial cells exposed to elexacaftor/tezacaftor/ivacaftor suggest benefits beyond improved CFTR channel function

**DOI:** 10.1152/ajplung.00272.2024

**Published:** 2024-10-22

**Authors:** Thomas H. Hampton, Roxanna Barnaby, Carolyn Roche, Amanda Nymon, Kiyoshi Ferreira Fukutani, Todd A. MacKenzie, Lily A. Charpentier, Bruce A. Stanton

**Affiliations:** ^1^Department of Microbiology and Immunology, Geisel School of Medicine at Dartmouth, Hanover, New Hampshire, United States; ^2^Department of Biomedical Data Science, Geisel School of Medicine at Dartmouth, Lebanon, New Hampshire, United States

**Keywords:** airway cells, cystic fibrosis, gene expression, modulator therapy, Trikafta

## Abstract

The combination of elexacaftor/tezacaftor/ivacaftor (ETI, Trikafta) reverses the primary defect in cystic fibrosis (CF) by improving CFTR-mediated Cl^−^ and HCO_3_^−^ secretion by airway epithelial cells (AECs), leading to improved lung function and less frequent exacerbations and hospitalizations. However, studies have shown that CFTR modulators like ivacaftor, a component of ETI, have numerous effects on CF cells beyond improved CFTR channel function. Because little is known about the effect of ETI on CF AEC gene expression, we exposed primary human AEC to ETI for 48 h and interrogated the transcriptome by RNA-seq and qPCR. ETI increased CFTR Cl^−^ secretion, and defensin gene expression (*DEFB1*), an observation consistent with reports of decreased bacterial burden in the lungs of people with CF (pwCF). ETI decreased *MMP10* and *MMP12* gene expression, suggesting that ETI may reduce proteolytic-induced lung destruction in CF. ETI also reduced the expression of the stress response gene heme oxygenase (*HMOX1*). qPCR analysis confirmed *DEFB1*, *HMOX1*, *MMP10*, and *MMP12* gene expression results observed by RNA-seq. Gene pathway analysis revealed that ETI decreased inflammatory signaling, cellular proliferation, and MHC class II antigen presentation. Collectively, these findings suggest that the clinical observation that ETI reduces lung infections in pwCF is related in part to drug-induced increases in *DEFB1* and that ETI may reduce lung damage by reducing *MMP10* and *MMP12* gene expression. Moreover, pathway analysis also identified several other genes responsible for the ETI-induced reduction in inflammation observed in pwCF.

**NEW & NOTEWORTHY** Gene expression responses by CF AECs exposed to ETI suggest that in addition to improving CFTR channel function, ETI is likely to enhance resistance to bacterial infection by increasing levels of beta-defensin 1 (hBD-1). ETI may also reduce lung damage by suppressing MMP10 and MMP12 and reduce airway inflammation by repressing proinflammatory cytokine secretion by CF AECs.

## Graphical abstract

**Figure:**
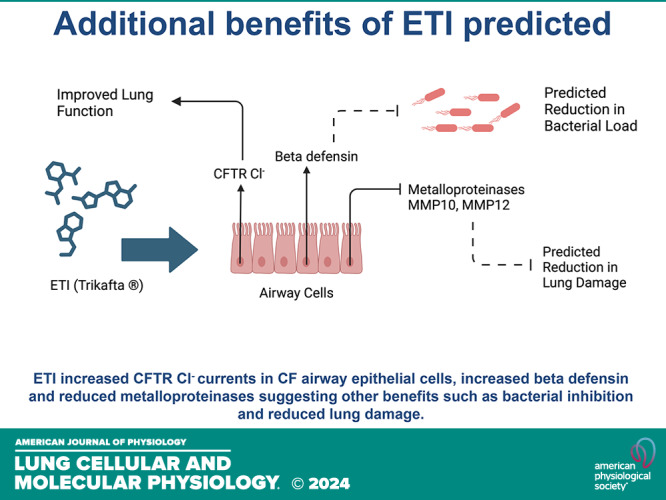

## INTRODUCTION

Cystic fibrosis (CF) is a recessive genetic disease caused by mutations in the gene that codes for the cystic fibrosis transmembrane conductance regulator, CFTR (1). Mutations in the *CFTR* gene lead to chronic bacterial lung infections, prolonged and excessive inflammation, and a progressive decrease in lung function (1, 2). Several drugs have been developed for people with CF (pwCF), including Kalydeco, Orkambi, and Symdeko (3). Kalydeco (ivacaftor) improves CFTR function and clinical outcomes for class III channel**-**gating mutations (e.g., G551D), Orkambi, the combination of ivacaftor and lumacaftor (4), improves CFTR function and clinical outcomes of people homozygous for the ΔF508 mutation, and Symdeko (tezacaftor and ivacaftor) improves CFTR function and clinical outcomes of people homozygous for the ΔF508 mutation, and for pwCF with one copy of ΔF508 and a mutation in CFTR responsive to Symdeko (5). Trikafta (elexacaftor, tezacaftor, and ivacaftor; ETI), approved by the Food and Drug Administration in 2019, is approved for pwCF having at least one copy of the ΔF508 mutation, the most common mutation that causes CF (6). In clinical trials, Trikafta has been shown to be more effective than Orkambi and Symdeko in improving lung function and reducing exacerbations and hospitalizations (6–9). Although the effects of ETI on Cl^−^ secretion by mutant CFTR have been investigated (10), there are few reports describing the effect of ETI on comprehensive gene expression by CF AECs (11, 12). Thus, the goal of studies in this report is to increase our knowledge of the effects of ETI on gene expression in human primary CF AECs.

Gene expression studies of blood samples drawn from pwCF have demonstrated that ivacaftor induces gene expression patterns associated with a more robust immune response (13), that subjects with the largest gene expression responses to ivacaftor experience the greatest clinical benefit (14), and that the combination of ivacaftor and lumacaftor decreased the expression of cell-death genes, *MMP9* and *SOCS3* (15). Although transcriptional responses in blood are relevant to CF, they do not capture responses in CF lung epithelial cells, and much less is known about how lung epithelial cells respond to modulator therapy at the transcriptional level. A transcriptional study of the IB3-1 CF line exposed to ivacaftor or lumacaftor reported no significant impact on immune gene signaling (16). Recently, a single-cell transcriptomic analysis of nasal swabs in children with CF showed that ETI partly restored interferon signaling in epithelial cells to levels seen in controls (11). In addition, ETI reduced the inflammatory phenotype of immune cells (11). A recent study of CF and wild-type airway epithelial exposed to ETI for 72 h reported many transcriptional differences between CF and wild-type cells but no response to ETI (12). Nasal epithelial transcriptomic profiles have recently been shown to predict changes in FEV_1_ (forced expiratory volume in 1 s) and BMI (body mass index) with ETI treatment (17). However, little is known about the response of CF AECs to ETI. An improved understanding of ETI-induced changes in gene expression in CF AECs will provide important information for future drug development efforts in CF.

To address this knowledge gap, we exposed human primary AECs from five CF donors in air-liquid interface (ALI) culture to ETI for 48 h and measured gene expression by RNA-seq as described in methods. We found that 11 genes were differentially expressed by ETI, including *DEFB1*, *HMOX1*, *MMP10*, and *MMP12*. Transcriptional changes in gene expression were validated by qPCR. Gene pathway analysis revealed that ETI decreased inflammatory signaling, cellular proliferation, and MHC class II antigen presentation. Collectively, the data suggest that the ETI-induced reduction in lung infections in pwCF are related in part to drug-induced increases in *DEFB1*, and that ETI may reduce lung damage by reducing *MMP10* and *MMP12* gene expression, which is predicted to reduce matrix metalloprotease activity. Moreover, pathway analysis also identified several genes responsible for the ETI-induced reduction in inflammation observed in pwCF.

## METHODS

### Cell Culture and ETI Exposure

Deidentified primary human AECs from five CF donors homozygous for the ΔF508 mutation were obtained from Dr. Scott Randell (University of North Carolina at Chapel Hill, Chapel Hill, NC) grown in ALI culture as previously described and tested for mycoplasma contamination (18, 19). RNA-seq data confirmed that the cells were primary airway epithelial cells. One of the donors was male and four of the donors were females. Donors were between 14 and 27 yr old and all were nonsmokers. The *n* in this report represents biological replicates. In brief, AECs were grown in BronchiaLife basal medium (Lifeline Cell Technology, Frederick, MD, Cat. No. LM-0007) supplemented with the BronchiaLife B/T LifeFactors Kit (Lifeline Cell Technology, Cat. No. LS-1047) and penicillin (10,000 U/mL) and streptomycin (10,000 μg/mL) (Sigma-Aldrich, Cat. No. P4333). Cells were studied between passages 4–9 and results were independent of passage number. The Dartmouth Committee for the Protection of Human Subjects determined that the use of CF AECs in this report is not considered human subject research because cells are taken from discarded tissue and contain no patient identifiers. CF AECs (500,000) were seeded on 24-mm permeable supports (Corning, Corning, NY, Cat. No. 3407), coated with Collagen type IV (Sigma-Aldrich, Cat. No. C-7521), and grown in an ALI media (University of North Carolina, Chapel Hill, NC) at 37°C for 3–4 wk to establish polarized monolayers (20, 21). Ivacaftor (10 nM) (Selleck Chemicals, Houston, TX, Cat. No. S1144), elexacaftor (3 µM) (Selleck Chemicals, Houston, TX, Cat. No. S8851), and tezacaftor (3 µM) (Selleck Chemicals, Houston, TX, Cat. No. S7059) or vehicle (DMSO) (Fisher Scientific, Hampton, NH, Cat. No. D128-500) were added to the basolateral media 48 h before experiments. ETI was added for 24 h, media was removed, and fresh media with ETI was added back for an additional 24 h. The total exposure to ETI was 48 h.

After 48 h of exposure to ETI or vehicle (DMSO), polarized CF AECs were rinsed three times with PBS ++ (Invitrogen, Waltham, MA, Cat. No. 14190-250), and RNA was isolated by the miRNeasy kit (Qiagen, Germantown, MD, Cat. No. 217004). cDNA synthesis was performed using RNA (1 µg) as input using SuperScript IV (Invitrogen, Grand Island, NY, Cat. No. 18090050). qPCR was performed using TaqMan Master Mix (Invitrogen, Waltham, MA, Cat. No. 4304437) as recommended by the manufacturer.

### RNA-Seq

Total RNA was isolated using the miRNeasy kit (Qiagen, Germantown, MD, Cat. No. 217004). RNA for RNA-seq was quantified by Qubit and integrity measured on a fragment analyzer (Agilent). RNA (100 ng) was used as input into the Quantseq FWD workflow (Lexogen) for library preparation following manufacturer’s instructions. Libraries were pooled for sequencing on a NextSeq2000 instrument targeting 10 M, single-end 100 bp reads per sample. Paired-end reads were trimmed using cutadapt v4.0 (22), and aligned to the human genome reference GRCh38, using Hisat2 v2.1.0, RRID:SCR_015530m (23). Genes were quantified using FeatureCounts v2.0.1 (24), with Human Ensembl v97 as the gene annotation reference. Alignment and quantification metrics were calculated using Samtools v1.15.1, RRID:SCR001105 (25).

### Filtering and Normalization

Filtering and normalization were performed in edgeR version 4.0.1 (26) as follows. Genes with fewer than 10 counts in any given sample or fewer than 15 counts across all 10 samples were filtered out using the filterByExpr function, leaving 15,229 genes for downstream analysis. Library sizes were adjusted using calcNormFactors and normalized log base 2 expression values were retrieved using the cpm function in edgeR.

### Exploratory Data Analysis

Euclidean distances between samples were calculated using the dist function in base R and an adonis test was performed using adonis2 in the R package vegan (27) and visualized using fviz_pca_ind in package factoextra 1.0.7. (28).

### Differential Gene Expression

Differential gene expression analysis of RNA-seq data was performed using genewise negative binomial generalized linear models in edgeR version 4.0.1 (26). Fastq files for all RNA-seq samples, as well as count tables of raw and normalized aligned reads, have been deposited in NCBI’s Gene Expression Omnibus (29) GSE268718 (https://www.ncbi.nlm.nih.gov/geo/query/acc.cgi?acc=GSE268718).

### Ingenuity Pathway Analysis

Significantly activated or repressed gene pathways were identified using QIAGEN IPA, RRID:SCR_008653, (QIAGEN Inc., https://digitalinsights.qiagen.com/IPA) as follows. ENSEMBL gene identifiers RRID:SCR_002344, log base 2 fold change responses to ETI compared with DMSO, and unadjusted *P* values (Supplemental Dataset S1) were uploaded to IPA and mapped to the Ingenuity knowledge base, and a new core analysis was created. Significantly differentially expressed genes for this analysis were defined as those with an absolute log2 fold change > 1 and an unadjusted *P* value < 0.05, resulting in 281 analysis-ready genes of which 178 were repressed and 103 were induced. Fisher’s exact tests of gene set enrichment performed by IPA on 537 Ingenuity canonical pathways identified seven pathways with an absolute activation z score > 1 and a *P* value < 0.05 and relevant to AECs.

### Measurement of Gene Expression by qPCR

Quantitative polymerase chain reaction (qPCR) was used to confirm significant ETI effects observed in RNA-seq analysis for *HMOX1*, *DEFB1*, *MMP10,* and *MMP12* and to explore the possibility that qPCR might detect significant changes in *IL1B* and *TNF*, two genes not significantly differentially expressed (FDR < 0.05) in RNA-seq, but relevant to the airway immune response in CF (30, 31). In addition, ETI responses were measured by qPCR in candidate reference genes *GAPDH*, *GUSB*, *HPRT1*, *HSP90AB,* and *UBC*.

Triplicate measurements of cycle threshold (CT) of a given gene on the same PCR plate were averaged to create a matrix of CT values for each target and reference gene in each condition (Supplemental Dataset S2). The three most stable reference genes (*HSP90AB*, *GAPDH*, and *HPRT1*) were identified by the geNorm2 function in the R package ctrlGene (32). Mixed-effect linear models in lme4_1.1–35.3 (33) and *P* values were generated using lmerTest_3.1–3 (34) to estimate the response to ETI, using the average CT of *HSP90AB, GAPDH, HPRT1* to control for differences in sample quality unrelated to treatment, modeling airway cell donor as a random effect (18).

### Measurement of Cytokines and Beta-Defensin 1 by ELISA

Secretion of hBD-1 (PeproTech: #900-K202), IL-6, IL-8, G-CSF, and MCP1 (R & D Systems: #DY206, #DY208, #DY214, #DY279) by CF AECs exposed to ETI or vehicle was measured by ELISA in triplicate wells per sample as recommended by R&D Systems. Concentrations of analytes measured by ELISA were calculated from standard curves of concentration as a function of fluorescence fitted with a four-parameter model using the R drm function in the drc 3.0–1 package (35). The statistical significance of ETI effects was assessed based on one-tailed *t* tests of log base2 fold changes. The alternative hypothesis of these tests was contingent on the direction of change of the corresponding gene measured by RNA-seq. For example, if the gene corresponding to beta-defensin 1 (*DEFB1*) was induced, then the alternative hypothesis for the test was “greater.”

### Measurements of ΔF508 CFTR Cl^−^ Currents in Primary CF AEC

Primary CF AECs were exposed to ETI or vehicle as described above for 48 h. To measure ΔF508 CFTR Cl^−^ secretion, AECs from the same donors and the same passage as described above, on Snapwell filters were mounted in Ussing chambers (Physiologic Instruments, San Diego, CA) whereupon the transepithelial voltage was clamped to 0 mV, and the short circuit current (Isc) was measured as described previously (1, 19, 20, 36). Briefly, amiloride (50 μM) was added to the apical solution to inhibit sodium reabsorption and then ΔF508 CFTR Cl^−^ secretion was stimulated with forskolin (10 μM; Sigma-Aldrich), followed by VX-770 (Ivacaftor; 5 μM, Selleckchem, Houston, TX). Finally, thiazolidinone (CFTRinh-172, 20 μM; Millipore, Billerica, MA) was added to the apical solution to inhibit ΔF508 CFTR Cl^−^ secretion. Data are expressed as the CFTRinh-172 inhibited Isc, which is presented as μA/cm^2^. Data were analyzed using the Data Acquisition Software Acquire and Analyze (Physiologic Instruments, San Diego, CA).

### General Statistical Analysis

Data were analyzed using the R statistical platform (37). Figures were created using ggplot2, RRID:SCR_014601 (38). The figure legends describe the specifics of the statistical analysis of each dataset and include the precise *P* values. *n* represents the number of donors. R code used for data analysis is available in GitHub: https://github.com/DartCF/ETI_Analysis.

## RESULTS

### ETI Increases ΔF508 CFTR Cl^−^ Currents in Primary CF AEC

**Figure 1.**
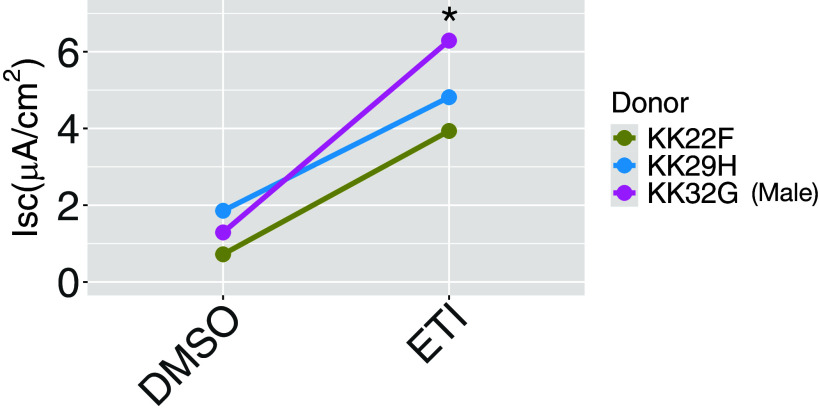
Short circuit current measurements following 48 h exposure to ETI in AECs from three donors. ETI increased ΔF508 CFTR Cl^−^ currents (*P = 0.028, mixed-effect linear model with donor as a random effect) compared with DMSO control. Donors KK22F and KK29H are female, and KK32G (purple) is male. AEC, airway epithelial cell; ETI, elexacaftor/tezacaftor/ivacaftor.

Human primary AECs in ALI culture obtained from three donors were exposed to ETI or vehicle for 48 h. Exposure to ETI increased ΔF508 CFTR Cl^−^ currents by 3.73 μA/cm^2^ (*P* = 0.028), a 289% increase compared with control CF AECs exposed to vehicle (Fig. 1).

### Differences between Donors Explain Most of the Divergence between Control and ETI Exposure

**Figure 2.**
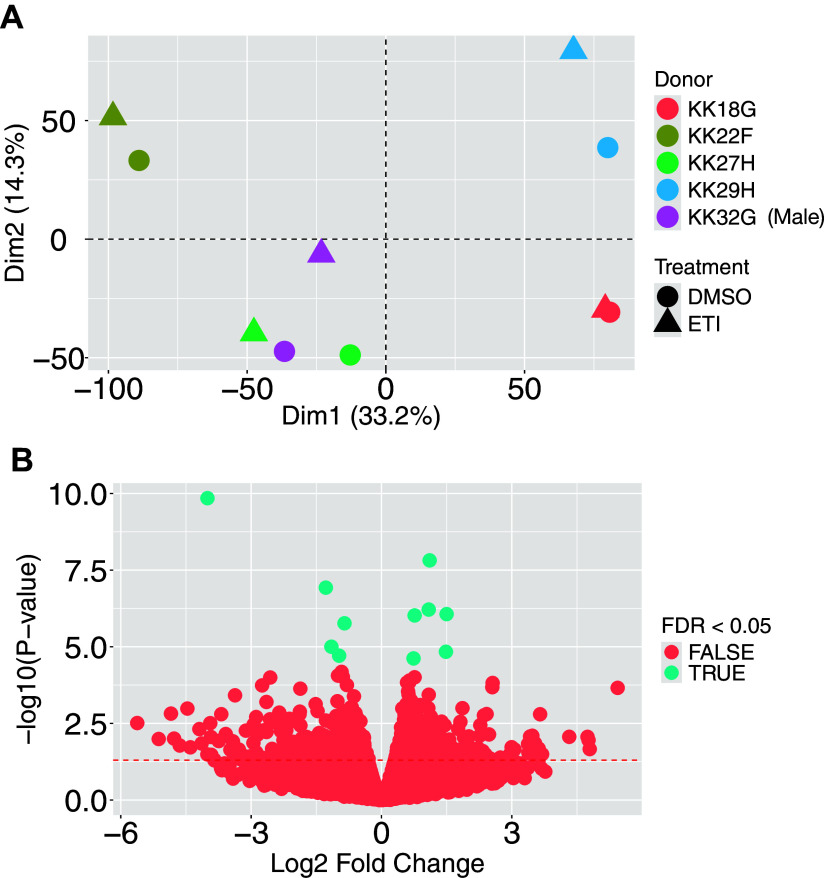
*A*: principal component analysis of AECs from five CF donors exposed to ETI or vehicle (DMSO) for 48 h. Numbers in parentheses indicate the percentage of variability explained by a given principal component (Dim=Dimension). Donor KK32G (purple) is male. *B*: volcano plot of gene expression. Eleven genes significantly affected by ETI (FDR <0.05) are indicated in turquoise. *CFTR* gene expression was not significantly affected by ETI (log2FC = −0.27; *P* = 0.44). AEC, airway epithelial cell; CF, cystic fibrosis; ETI, elexacaftor/tezacaftor/ivacaftor.

Human primary AECs in ALI culture obtained from five donors with CF were exposed to ETI or vehicle for 48 h. RNA was isolated, cDNA libraries were created, sequenced, aligned to a human reference genome, and normalized in edgeR as described in methods. Dimension reduction by principal components analysis (Fig. 2*A*) revealed that samples clustered closer together by donor rather than ETI treatment. An adonis test of Euclidean distances between the 10 samples verified that 70% of the variability between samples can be explained by differences between donors (*P* = 0.001) and only 6% of differences were explained by ETI treatment, although this effect did not reach significance (*P* = 0.35).

Eleven of the 15,283 genes identified with at least 10 counts in any given sample and at least 15 counts across all 10 samples were differentially expressed (FDR < 0.05) in ETI-exposed CF AECs compared with vehicle control as shown in Table 1 and graphically in Fig. 2*B*. It is important to note that others have reported that CFTR modulators like ivacaftor, ivacaftor and lumacaftor in combination, and ETI also have very modest effects on gene expression (11, 12, 39).

**Table 1.** Eleven genes significantly changed by ETI (FDR < 0.05) ordered by increasing fold change

Symbol	Entrez Gene Name	log2FC	*P* value	FDR
CYP2F1	Cytochrome P450 family 2 subfamily F member 1	−4	1.40E-10	2.20E-06
HMOX1	Heme oxygenase 1	−1.3	1.20E-07	6.00E-04
SPOCK2	SPARC (osteonectin), cwcv and kazal like domains proteoglycan 2	−1.2	1.00E-05	0.019
MMP12	Matrix metallopeptidase 12	−0.97	2.00E-05	0.03
MMP10	Matrix metallopeptidase 10	−0.85	1.70E-06	0.0038
RNASE1	Ribonuclease A family member 1, pancreatic	0.74	2.40E-05	0.033
MT-ND4L	NADH dehydrogenase subunit 4 L	0.76	9.50E-07	0.0024
SLC22A18	Solute carrier family 22 member 18	1.1	6.10E-07	0.0023
DEFB1	Defensin beta 1	1.1	1.50E-08	0.00012
RPS2P46	Ribosomal protein S2 pseudogene 46	1.5	8.60E-07	0.0024
MTND2P28	MT-ND2 pseudogene 28	1.5	1.50E-05	0.025

A log2 FC of one is a 200% change in gene expression. ETI, elexacaftor/tezacaftor/ivacaftor.

Nonetheless, several of the genes that were differentially expressed are intriguing in the context of the CF airway. For example, *DEFB1,* which was increased by ETI, codes for human beta-defensin 1, an antimicrobial peptide produced by AECs (40). *DEFB1* expression is activated in the response to airway pathogens (41), including *Pseudomonas aeruginosa* (42). Reduced pH in the CF airway surface liquid reduces DEFB1 activity (41). However, when modulators like ETI restore pH levels to near-normal levels (42), they likely restore hBD-1 (the protein encoded by *DEFB1*) activity. Moreover, increased *DEFB1* gene expression coupled with elevated protein is likely to reduce pathogen abundance and contribute to ETI’s remarkable success (6–9, 42).

Matrix metallopeptidases have a well-established role in CF lung disease as they degrade connective tissue and lead to irreversible lung damage (43), making them attractive targets for drug development (44). Therefore, the observed decrease in the expression of the matrix metallopeptidase genes *MMP10* and *MMP12* by ETI, suggest that ETI may slow lung damage in CF.

Finally, ETI treatment reduced the expression of the stress response gene heme oxygenase (*HMOX1*). Heme oxygenase breaks heme down and releases carbon monoxide (CO), iron, and biliverdin (45). These byproducts promote anti-inflammatory signaling in the CF airway, as reviewed by DiPietro et al. (46) and CO is bactericidal against *Pseudomonas aeruginosa* (47). Indeed, *HMOX1* is induced in response to *Pseudomonas* and is a known modifier gene of CF expression (48). Notably, expression of *HMOX1* is reduced in CF (49). Therefore, decreased *HMOX1* gene expression in CF AECs following ETI suggests that ETI might compromise an antibacterial and anti-inflammatory state that is already deficient in CF.

### qPCR Confirmation of ETI-Induced Changes in Genes Observed by RNA-Seq

**Figure 3.**
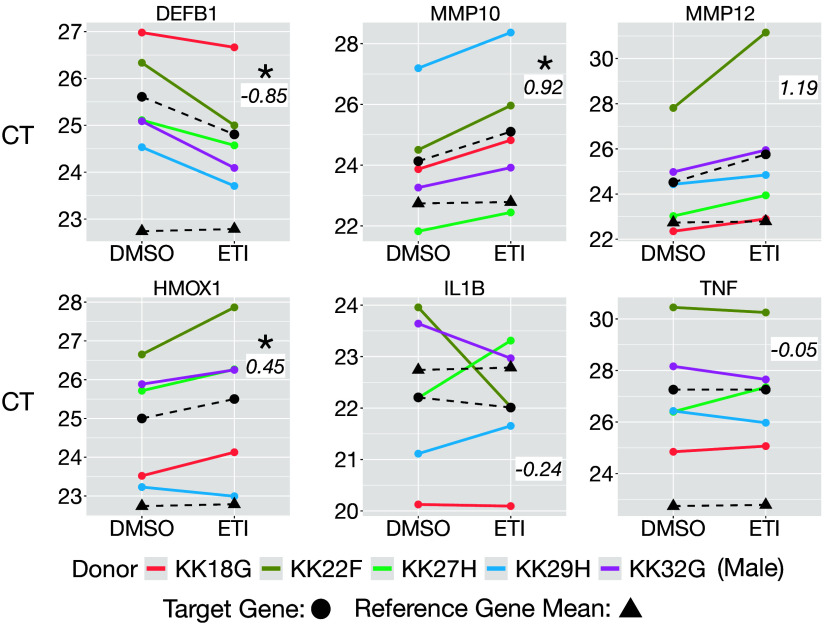
qPCR measurements of *DEFB1, MMP10, MMP12, HMOX1, IL1B, TNF,* and reference genes. Cycle threshold (CT) mean values of each target gene (black circles connected by a dashed line) and reference gene mean values (black triangles connected by a dashed line) are presented. CT values of the gene of interest are shown for each donor (colors). The figure shows that the average of the three reference genes (*HSP90AB, GAPDH,* and *HPRT1*) was neither induced nor repressed in response to ETI: the slope of dashed lines connecting the mean of reference genes in the two conditions is close to zero. *DEFB1, MMP10, MMP12,* and *HMOX1* were significantly differentially expressed in response to ETI using a mixed-effect linear model of the ETI effect on each target gene accounting for the mean of the reference genes with donor modeled as a random effect. *DEFB1*: *P* = 0.02, *MMP10*: *P* = 0.03, *MMP12*: *P* = 0.09, *HMOX1*: *P* = 0.02, IL1B: *P* = 0.79, TNF: *P* = 0.99. Delta-delta CT values for each target shown in italics in each panel. *P < 0.05. A positive ΔΔCT value of 1 corresponds to a twofold (200%) increase in abundance in the gene of interest. Donor KK32G (purple) is male. ETI, elexacaftor/tezacaftor/ivacaftor.

qPCR was performed to validate significant changes in gene expression observed using RNA-seq. Reassuringly, *DEFB1*, *MMP10*, *MMP12*, and *HMOX1*, which were differentially expressed as determined by RNA-seq (FDR < 0.05), were also differentially expressed as assessed by qPCR (Fig. 3). *IL1B* and *TNF,* two highly relevant immune genes in CF, were not significantly differentially expressed by ETI exposure as determined by both RNA-seq and qPCR.

### Effect of ETI on hBD-1, G-CSF, IL-6, IL-8, and MCP1 Secretion by Primary CF AECs

**Figure 4.**
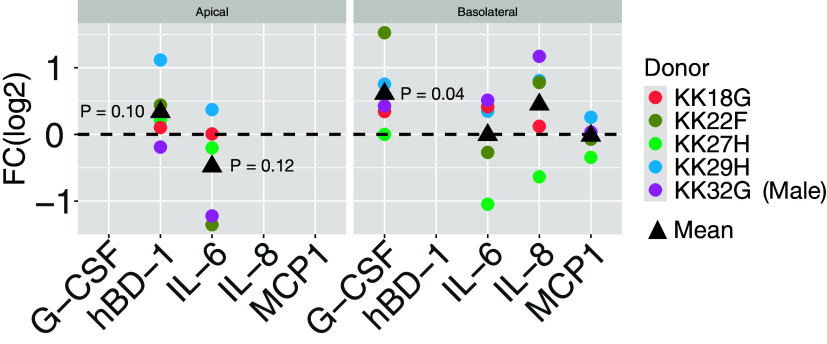
Response to ETI measured as log base 2 fold change [FC(log2)] for beta defensin 1 (hBD-1) and cytokines detected by ELISA in apical (*left*) or basolateral fluid (*right*) for each donor (colors) and the mean of all donors (black triangles). Significance was assessed by one-tailed *t* tests in which the alternative hypothesis was selected based on prior RNA-seq measurements. Exact significance is annotated for tests with *P* < 0.2. There was sufficient fluid on the apical side of AECs of the air-liquid interface (ALI) culture to measure hBD-1 and IL-6, but not other analytes. Donor KK32G (purple) is male. ETI, elexacaftor/tezacaftor/ivacaftor.

High-throughput measurement of gene expression readily captures the expression of most genes, but changes in protein expression are more likely to predict changes in phenotype, and often gene expression does not correlate with protein expression. For this reason, we used ELISA to assess protein responses to ETI in the same samples that were assessed by RNA-seq and qPCR. Samples were interrogated for several proteins relevant to CF, including hBD-1, G-CSF, IL-6, IL-8, and MCP1. As shown in Fig. 4, changes in beta-defensin 1 (hBD-1, the protein coded by *DEFB1*) in apical supernatants of CF AEC exposed to ETI increased, but the increase was not significant (Table 2). It is important to note that some donors appeared to respond to ETI, similar to the variable response of pwCF exposed to ETI (6, 11, 17, 50). For example, ETI increased hBD-1 by 1.12-fold (log_2_) in donor KK29H (turquoise color), a >200% increase above vehicle control. On the other hand, ETI exposure had a much smaller impact on hBD-1 secretion in donors like KK32G (pink color), where hBD-1 levels were at or below baseline levels.

**Table 2.** ETI effect on expression of genes shown in Fig. 5

Symbol	Entrez Gene Name	log2FC	*P* value
*Inflammation*			
CTSV	Cathepsin V	−1.1	0.048
HLA-DMB	Major histocompatibility complex, class II, DM beta	−2.1	0.0022
HLA-DPB1	Major histocompatibility complex, class II, DP beta 1	−2.6	0.043
IL6	Interleukin 6	−1.6	0.0097
KIF2C	Kinesin family member 2 C	−3.3	0.03
*Drug Metabolism*			
CHST2	Carbohydrate sulfotransferase 2	−1.1	0.0031
CHST4	Carbohydrate sulfotransferase 4	−1.4	0.049
CYP1A2	Cytochrome P450 family 1 subfamily A member 2	−3.5	0.023
HS6ST2	Heparan sulfate 6-O-sulfotransferase 2	−1.1	0.018
*Proliferation*			
CCNA2	Cyclin A2	−2.9	0.028
CDC26	Cell division cycle 26	−2.3	0.011
CDC6	Cell division cycle 6	−2.3	0.0057
CDT1	Chromatin licensing and DNA replication factor 1	−2.9	0.0035
CEP131	Centrosomal protein 131	−1.3	0.018
FBXL7	F-box and leucine rich repeat protein 7	−1.3	0.012
H2BC9	H2B clustered histone 9	−2.3	0.039
MYBL2	MYB proto-oncogene like 2	−2.5	0.035
*Multiple*			
PPP2R3B	Protein phosphatase 2 regulatory subunit B'beta	−1.3	0.013

ETI, elexacaftor/tezacaftor/ivacaftor.

Donor-to-donor variability was also observed in the secretion of G-CSF, IL-6, IL-8, and MCP1 in the basolateral media in response to ETI (Fig. 4). Only G-CSF, which enhances neutrophil-mediated immunity (51) was significantly increased by ETI (*P* = 0.04). G-CSF was detected in basolateral supernatants (Fig. 4, *right*) and its response to ETI was concordant with gene expression responses, with an average increase in G-CSF of 150% (*P* = 0.04). Donor KK22F (olive) showed the greatest increase in basolateral G-CSF, achieving a 288% increase relative to control.

### Gene Pathway Analysis by Ingenuity Pathway Analysis

The preceding sections focused on the behavior of individual genes that were significantly altered by ETI and the proteins the genes encode. However, groups of genes (e.g., genes belonging to the same biological pathway), work together to maintain homeostasis and respond to pathogens and drugs. We therefore hypothesized that exposing AECs to ETI might target genes in specific biological pathways more than expected by chance, especially if we relax our definition of significance to include any gene with a nominal *P* value less than 0.05 and an absolute log2 fold change greater than 1, as is customary in pathway analysis (52, 53). This hypothesis is readily tractable using overrepresentation analysis in Ingenuity Pathway Analysis (IPA: QIAGEN Inc., https://digitalinsights.qiagen.com/IPA) by Fisher’s exact test. IPA identifies gene pathways in which an experimental condition (e.g., ETI) affects the expression of multiple genes in canonical gene pathways. Achieving significance in overrepresentation on a particular path does not require that every gene in the pathway is significantly changed by the treatment, but rather that a significantly higher proportion of differentially expressed genes belong to the pathway than would be predicted by chance.

**Figure 5.**
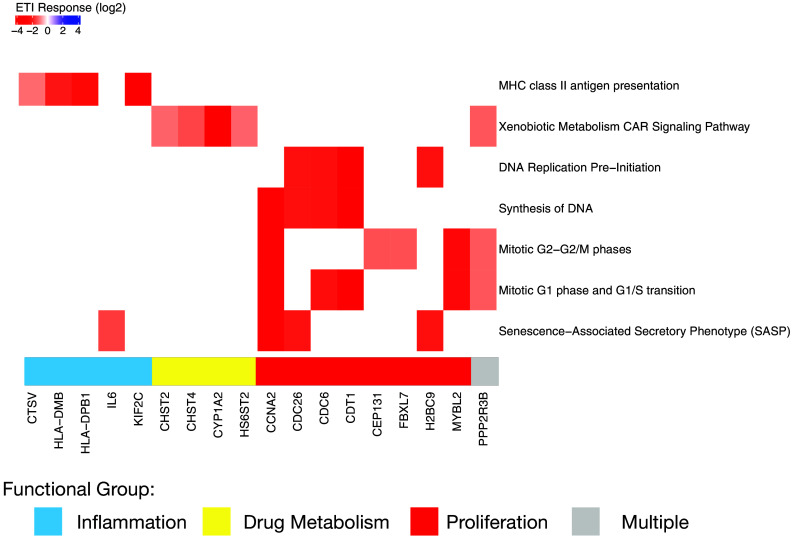
IPA canonical pathways that are relevant to AECs. All pathways in the figure are repressed (absolute z-score > 1), and significantly enriched (*P* < 0.05, Fisher’s exact test). Pathway names are shown on the right and the gene symbols of genes mapping to each pathway that were differentially expressed are shown on the *x*-axis. Blanks (white color) indicate that a gene is not on a pathway. For example, cathepsin V (CTSV, far left) was significantly repressed by ETI and is a member of the MHC class II antigen presentation pathway, but not other pathways in this figure. AEC, airway epithelial cell; ETI, elexacaftor/tezacaftor/ivacaftor; IPA, Ingenuity Pathway Analysis.

The results of IPA analysis are summarized in Fig. 5, which shows the genes (columns) that participated in the identification of IPA canonical pathways (rows) that had a significantly higher proportion of differentially expressed genes than predicted by chance. Using a less restrictive definition of significance (nominal *P* < 0.05 and absolute log2 fold change greater than 1) 288 genes were used as input for overrepresentation analysis in IPA. One hundred and seventy-eight of these genes were repressed and 103 were induced in response to ETI. Figure 5 only includes pathways that were systematically biased toward induction or repression, that is, they had an IPA activation z score greater than 1 or less than −1.

Figure 5 illustrates three general features of the analysis. First, all of the data in Fig. 5 are colored red, meaning that differentially expressed genes mapping to targeted pathways were all repressed. Deeper red colors indicate greater repression in response to ETI. Second, a relatively small number of differentially expressed genes is responsible for pathway enrichment. For example, MHC class II antigen presentation in the top row of Fig. 5 was significantly enriched based on differential expression of four genes *CSTV*, *HLA-DMB*, *HLA-DBP1*, and *KIF2C*. Third, the same differentially expressed genes contribute to the significant enrichment of several pathways that are biologically related to each other, which are color coded at the bottom of Fig. 5 and described in Table 2.

### Decreased Inflammatory Signaling

ETI reduced inflammatory signaling by CF AECs as evidenced by significantly reduced expression (log2 fold change < 1, *P* < 0.05) of *CSTV*, *HLA-DMB*, *HLA-DBP1,* and *IL6*. The IL-6 cytokine was repressed in the apical media collected from AECs exposed to ETI, but the change was not significant (Fig. 4 and Table 2).

### Decreased Xenobiotic Response

ETI reduced *CHST2*, *CHST4*, *CYP1A2*, *HS6ST2*, and *PPP2R3B* on the xenobiotic metabolism CAR signaling pathway as shown in Fig. 5. AECs efficiently metabolize drugs (54) and it is possible that repressed xenobiotic metabolism could alter drug-drug interactions in the airway. Sufficient concentrations of drugs can trigger phase I and phase II detoxification processes in human AECs (55). Systematic repression of *CHST2*, *CHST4*, *CYP1A2*, *HS6ST2*, and *PPP2R3B* therefore argues against the hypothesis that ETI places a significant toxic burden on AECs.

### Decreased Cellular Proliferation

ETI decreased cell proliferation genes associated with DNA replication, synthesis, and mitotic phase transition (*CCNA*, *CDC26*, *CDC6*, *CDT1*, *CEP131*, *FBXL7*, *H2BC9*, *H2BC9*, *and MYBL2*), suggesting that ETI exposure decreases proliferation of CF AECs.

## DISCUSSION

Taken together, our analysis of primary human CF AECs from five donors reveals that ETI may improve patient outcomes in part by increasing ΔF508 Cl^−^ secretion, enhancing antibacterial activity, reducing lung damage, and suppressing proinflammatory signaling (Fig. 5 and Table 2). Collectively, the data suggest that the ETI-induced reduction in lung infections in pwCF is related in part to drug-induced increases in *DEFB1* and that ETI may decrease lung damage by reducing *MMP10* and *MMP12* gene expression, which is predicted to reduce matrix metalloprotease activity. Moreover, pathway analysis also identified several genes responsible for the ETI-induced reduction in inflammation observed in pwCF. Our data are consistent with literature reports that ETI reduces bacterial burden and inflammation in the CF airway (56), improves lung function, and reduces exacerbations and hospitalizations with few side effects (7, 9, 42, 56).

This study identifies mechanisms through which ETI is likely to improve antibacterial function, reduce lung damage, and reduce proinflammatory signaling. Specifically, we found that ETI increased gene expression of *DEFB1* and reduced the expression of genes mediating inflammation and lung damage. Beneficial effects of other CFTR modulators have been previously reported (13) that subjects with the largest gene expression responses to ivacaftor experience the greatest clinical benefit (14) and that the combination of ivacaftor and lumacaftor decreased the expression of cell-death genes, *MMP9* and *SOCS3* (15). We have shown previously that exposing human CF monocyte-derived macrophages to lumacaftor and ivacaftor reduced transferrin receptor 1 expression, corrects dysfunctional iron transport gene expression observed in CF (57), and that lumacaftor restores the ability of CF macrophages to phagocytose and kill *Pseudomonas* (58).

Two recent studies have examined the effect of ETI on the transcriptional response of nasal swabs obtained from pwCF (11, 17). Loske et al. (11) reported that ETI improves innate immunity and suppresses immune cell inflammatory responses in nasal epithelial cells obtained from children with CF. Yue et al. (17) identified inverse correlations between inflammatory gene expression (e.g., *TLR4* and *IL10*) and FEV1, but they did not find that *DEFB1*, *MMP10*, *MMP12*, *HMOX1* (genes we identified as responsive to ETI) were ideal biomarkers to predict clinical outcomes in pwCF. Transcriptional responses in patient-derived intestinal organoids exposed to ETI suggest that in the context of the gut, ETI induces biological processes related to chemokines and signaling, chemotaxis, and tissue development (59).

As noted above in Fig. 2*A*, most of the variation in the data is attributable to donor differences rather than ETI exposure. This is not surprising given that pwCF respond to ETI in varying degrees: some have robust responses and some have small or negligible responses (6, 7, 9, 42, 56). Moreover, many studies that have examined the effect of other modulators of CFTR have shown very small or no effects on gene expression (14, 15, 39). It should be noted that predictions based primarily on in vitro gene expression and ELISA analysis of cytokine secretion by CF AECs need to be validated by clinical measurements of cytokines in bronchoalveolar fluid (BALF) and/or sputum (if available) in pwCF. Several studies have shown in pwCF that ETI reduces *Pseudomonas* abundance and systemic inflammation (7, 60–64). However, the effect of ETI on gene expression in lung tissue of pwCF has, to the best of our knowledge, not been reported.

### DEFB1, ETI, and Lung Infection

Antimicrobial peptides like beta-defensins are effective against CF pathogens (65), are secreted by AECs, are present in CF bronchoalveolar lavage fluid (40), and are predictive of lung disease severity (66). Genetic polymorphisms in *DEFB1* have been proposed to explain differences in colonization by *P. aeruginosa* in pwCF (67). Beta defensin 1 is pH sensitive (41), thus enhancement of the ability of CFTR to secrete Cl^−^ and HCO_3_^−^ and increase airway surface fluid pH as well as increase in *DEFB1* expression as a result of ETI exposure is predicted to increase the ability of hBD-1 to kill bacteria. However, we note that in this study, the increase in hBD-1 protein was not statistically significant; thus, it is likely that ETI may increase hBD-1 efficacy primarily by increasing the pH of airway surface liquid. This suggestion is consistent with studies in pwCF demonstrating that ETI reduces bacterial load (68).

### Decreased MMP10 and MMP12 by ETI in the CF Lung Is Predicted to Reduce Lung Damage

MMPs are expressed in and secreted by AECs (69) and play a key role in extracellular matrix remodeling by degrading extracellular matrix components. A pathogenic role for matrix metalloproteinase 12 (MMP12) in lung disease is supported by multiple lines of evidence (70). MMPs stimulate proinflammatory cytokines, including TNFα (71), and exacerbate chronic lung infection and inflammation by promoting tissue damage and fibrosis (72). MMP12 is upregulated in βENaC-Tg mice and contributes to early lung damage (70). MMPs also cleave inactive proforms of cytokines, such as pro-IL-1β, into their active forms, thereby potentiating inflammation (73). *P. aeruginosa*-induced increases in IL-6, TNFα, and IL-8 upregulate MMP-12 and thereby promote a proteolytic environment that facilitates lung destruction (72). Thus, a reduction in MMP10 and MMP12 by ETI in the CF lung is predicted to reduce the initiation and progressive nature of lung damage in CF.

Matrix metalloprotease MMP10 is also upregulated in primary human AECs exposed to *P. aeruginosa* and by airway pathogens in general (74–76) and in a mouse model of *P. aeruginosa* lung infection (74). *Mmp10*^−/−^ mice are more susceptible to pneumonia than wild-type mice (74). MMP10 facilitates the activation of macrophages from proinflammatory (M1-like) cells into immunosuppressive (M2-like) cells (77). ETI-mediated reduction of MMP10 is therefore also predicted to benefit people with CF.

### Decreased Inflammatory Signaling Induced by ETI

ETI reduced the expression of a cluster of genes with shared proinflammatory function (*CSTV*, *HLA-DMB*, *HLA-DBP1*, and *IL6*) (Fig. 5), consistent with other studies demonstrating that ETI reduces inflammation in pwCF (78). A reduction in inflammation mediated by ETI will reduce immune cells in the lungs of pwCF and thereby reduce damage induced by, for example, neutrophils (79). ETI has been shown to reduce inflammation and cytokines in pwCF (11, 79), consistent with our studies on primary CF AECs. Our results also confirm many studies that cytokine secretion by AECs plays an important role in inflammation and lung damage in CF (30, 80–82).

### Modest Gene Expression Responses to ETI Are Similar to Previous Studies with Other Modulators of Mutant CFTR

Both ETI and ivacaftor are often referred to as “highly effective modulator therapy” because they dramatically improve CF lung function and patient outcomes. Notably, ivacaftor is highly effective for the subset of pwCF who have qualifying gating mutations in CFTR, for example, G551D, whereas ETI, which itself contains ivacaftor, extends highly effective therapy to pwCF with the most common CFTR mutation, ΔF508, and a second responsive mutation. However, neither ETI nor ivacaftor seem to alter gene expression dramatically. Ivacaftor has many beneficial effects on patient outcomes (83–86) and rescues channel function (87). A recent study of the combination of ivacaftor and lumacaftor (Orkambi) identified only 36 genes that were differentially expressed in blood samples in response to ETI (15). Ivacaftor alone significantly affected 49 genes in a similar study of peripheral blood monocytes using FDR < 0.05 (13). In the same way, there is abundant evidence that ETI has many beneficial effects on patient outcomes (6–9) and rescues channel function (10) yet recent studies of ETI’s effect on gene expression suggest modest effects. Yue et al. (17) reported 136 differentially expressed genes at FDR < 0.05 in nasal epithelia exposed to ETI. CF organoids exposed to ETI identified 85 upregulated with ETI and 55 genes were significantly downregulated (FDR value < 0.1). Of these, only nine had a fold change response greater than 2 (59).

### The Current Study Has Several Strengths

To our knowledge, this is the first study to comprehensively examine the effect of ETI on primary human CF AECs in air-liquid interface culture. Our results are in general agreement with other studies on blood and nasal swabs that ETI reduces inflammation. In addition, we identified several genes, including *DEFB1*, *MMP10*, *MMP12*, *HMOX1,* and several proinflammatory immune pathways that are downregulated by ETI. Using qPCR, we confirmed the differential expression of these genes identified by RNA-seq. Notably, as suggested previously (18), we identified several reference genes that when averaged were not affected by ETI, and we presented the data on the CT values of reference genes as well as our genes of interest to demonstrate that the changes in ΔΔCT were due to changes in genes of interest as opposed to fluctuations in reference genes (18). Moreover, our studies were conducted on primary cultures of human CF AECs in air-liquid interface culture from five donors. Given the variability of the response of pwCF to ETI, we suggest that our studies on primary cells from five donors are likely to be more representative of the disease than studies that interrogate cell lines that have been obtained from a single donor where the *n* represents technical replicates from one biological donor, and the cells were obtained from tumors or have been genetically manipulated.

### We Also Acknowledge Limitations

First, this study used primary cells from five CF donors. Using additional donors would likely identify smaller changes in gene and/or cytokine expression as significant that failed to achieve significance in our study. For example, power analysis of our data revealed that ∼17 CF donors would be needed to achieve significance (*P* = 0.05, with 80% power) for proteins like IL-6 and hBD-1 that were affected by ETI but did not quite reach statistical significance (Fig. 4). Second, as with all studies of primary AECs obtained from pwCF, some donors may have been previously exposed to CFTR modulators, other medications, or other environment factors that may alter their response to ETI and therefore may account for some of the observations obtained in this study. Third, it is possible that other cell types respond to ETI differently from AECs, including immune cells, and the immune cell response to ETI may affect AEC gene expression in response to ETI. Future single-cell RNA-seq studies, beyond the scope of this report, might be useful to resolve cell-dependent effects of ETI. Importantly, our results are in general agreement with studies showing that ETI reduces *Pseudomonas* abundance and systemic inflammation (7, 60–64), so we believe that our findings on CF AECs may partly explain ETI’s outstanding efficacy.

### Conclusions

Our study of gene expression responses by CF AECs exposed to ETI suggests that in addition to improving CFTR channel function, ETI is likely to increase resistance to bacterial infection, and thereby account for the modest reduction in lung bacteria by increasing hBD-1, reducing lung damage by suppressing MMP10, and decreasing inflammation by repressing proinflammatory cytokine secretion. Furthermore, the reduction in proinflammatory cytokines is predicted to reduce inflammation by decreasing the recruitment of immune cells, which are known to induce lung damage, into the lungs.

## DATA AVAILABILITY

Data have been deposited in NCBI Gene Expression Omnibus (GSE268718; https://www.ncbi.nlm.nih.gov/geo/query/acc.cgi?acc=GSE268718).

## SUPPLEMENTAL MATERIAL

10.6084/m9.figshare.25975324.v1 Supplemental Dataset S1: https://doi.org/10.6084/m9.figshare.25975324.v1.

10.6084/m9.figshare.25997890.v1 Supplemental Dataset S2: https://doi.org/10.6084/m9.figshare.25997890.v1. 

## GRANTS

This work was supported by funding from the Cystic Fibrosis Foundation (STANTO19G0, STANTO20P0, and STANTO19R0), from the National Institutes of Health (P30DK117469 and R01HL151385), and the Flatley Foundation.

## DISCLOSURES

No conflicts of interest, financial or otherwise, are declared by the authors.

## AUTHOR CONTRIBUTIONS

T.H.H. and B.A.S. conceived and designed research; R.B., A.N., C.R., and L.A.C. performed experiments; T.H.H. and K.F.F. analyzed data; T.H.H., T.A.M., and B.A.S. interpreted results of experiments; T.H.H. prepared figures; T.H.H. and B.A.S. drafted manuscript; T.H.H., R.B., L.A.C., and B.A.S. edited and revised manuscript; T.H.H., R.B., A.N., C.R., K.F.F., T.A.M., L.A.C., and B.A.S. approved final version of manuscript.
